# Human papillomavirus subtypes distribution among 2309 cervical cancer patients in West China

**DOI:** 10.18632/oncotarget.16093

**Published:** 2017-03-10

**Authors:** Kemin Li, Rutie Yin, Danqing Wang, Qingli Li

**Affiliations:** ^1^ Department of Gynecology, West China Second University Hospital, Sichuan University, Chengdu, Sichuan, 610041, China; ^2^ Laboratory of Molecular Epidemiology of Birth Defects, West China Second University Hospital, Sichuan University, Chengdu, Sichuan, 610041, China

**Keywords:** cervical cancer, prevalence, human papillomavirus, distribution, West China

## Abstract

**Objective:**

The prevalence of cervical Human Papillomavirus (HPV) infection in cervical cancer varies greatly worldwide, and HPV prevalence and genotypes in China are limited. The objective is to analyze the prevalence of HPV subtypes in cervical cancer patients in west China as well as the relationships between different histological types of cervical cancer and HPV subtypes.

**Results:**

2309 cases were included. 90.86% were infected with HPV, and the remaining 9.13% were negative. The most common subtypes were HPV16, HPV18, HPV58, HPV53, and HPV33. HPV16 and HPV18 appeared to be the most prevalent HPV subtypes across all age groups, while the prevalence of the other subtypes varied between age groups. A logistic regression analysis revealed that the occurrence of cervical squamous cell carcinoma and adenocarcinoma was most closely correlated to a persistent infection with HPV16 or HPV18, with *P* < 0.05.

**Materials and Methods:**

The prevalence of 27 HPV subtypes in 2309 cervical cancer patients who received treatment at our hospital between June 2011 and January 2016 was analyzed based on the results of HPV testing using Liquid suspension chip technology (Luminex 200).

**Conclusions:**

HPV16 and HPV18 were the most prevalent HPV subtypes among the cervical cancer patients, followed by HPV58, HPV53, and HPV33. 9.13% of the cases appeared to not be associated with HPV. This suggests that HPV testing without the use of cytology may overlook some special types of cervical cancer that account for approximately 10% of all cervical cancer cases.

## INTRODUCTION

In 2015, China reported 98,900 new cases of cervical cancer and 30,500 cervical cancer deaths [1], as compared to the 12,900 new cases and 4100 deaths reported in the United States [2]. It is apparent that this cancer has become a serious threat to the lives and health of Chinese women. Numerous epidemiologic and laboratory studies have shown that persistent infection with high-risk human papillomavirus (HPV) types is the main cause of cervical cancer and cervical precancerous lesions [3–6]. Currently, HPV testing is one of the main methods of screening for cervical cancer and cervical precancerous lesions [7–10]. Additionally, cervical cytology has been widely used in screening for cervical cancer, and such tests have been shown to significantly reduce the incidence of cervical cancer. Nonetheless, studies have shown that the incidence of cervical cancer in China has increased annually and is occurring at a younger age. Because China is a developing country, many citizens are not aware of the importance of screening for cervical cancer, and many areas do not yet have universal cervical cancer screening.

Studies have found that the type-specific prevalence of HPV varies obviously by age and region. For example, in the southern part of China, HPV infections primarily occur among women under 30 years old. The HPV prevalence was estimated to be 26%, with 21.12% infected with high-risk HPV, and HPV52, HPV16, and HPV58 were the most prevalent subtypes [3]. In Yunnan Province, HPV and high-risk types were estimated to infect 38.1% and 32.4% of the local people, respectively. HPV16 was the most prevalent among all the HPV subtypes, followed by HPV52 and HPV58 [11].

The prevalence and HPV type also vary according to the grades of cervical cancer and cervical precancerous lesions. A study found that the prevalence of high-risk HPV was responsible for up to 89.8% of the invasive cervical cancer patients in Ghana. HPV18 appeared to be the most prevalent HPV subtype, and HPV59, HPV45, and HPV16 were the second most prevalent ones [6].

An analysis of the HPV subtypes of different ethnic groups in a country or a region is highly significant to prognosis evaluation, creating reexamination plans and clinical treatment plans, and developing preventive and therapeutic HPV vaccines. However, the HPV vaccine has not been widely administered in China. The HPV vaccine was not marketed in China until after 2016, and as a result, it has not been widely used. While HVP infections in healthy women and patients with cervical precancerous lesions have been reported in different provinces of China, there have been relatively few research reports on HPV infections among cervical cancer patients in China. Therefore, our research group analyzed HPV infections among cervical cancer patients that received treatment between June 2011 and January 2016 at the largest gynecological cancer prevention and treatment center in West China. Our aim was to provide a basis for cervical cancer prevention and treatment.

## RESULTS

**Figure 1:**
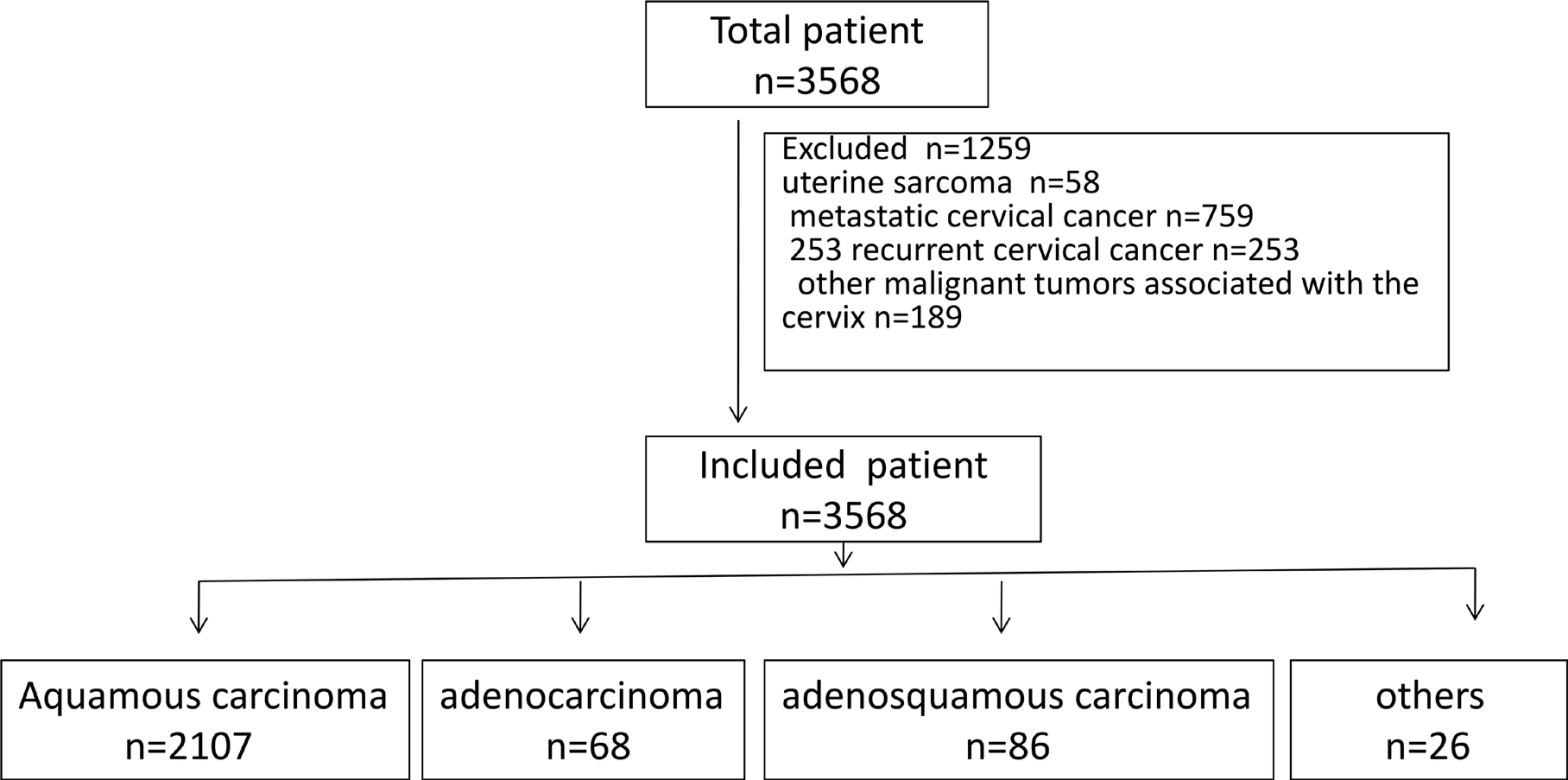
Overview of study population

Figure 1 display overview of study population. A total of 3568 cervical cancer patients, who received treatment at our hospital between June 2011 and January 2016, were evaluated for inclusion in this study. 1259 patients were excluded, including 58 uterine sarcoma, 759 metastatic cervical cancer, 253 recurrent cervical cancer, and 189 other malignant tumors associated with the cervix. A total of 2309 invasive cervical cancer patients were analyzed in this study. Table 1 summarizes their basic characteristics. The patients’ ages ranged from 21 to 81 years old, with an average of 45.36 years. Of the 2309cases, 2106 (91.21%) were squamous cell carcinomas, 86 (3.72%) were adenocarcinomas, and 68 (2.94%) were adenosquamous carcinomas. All of the cases were divided into the following five age groups: under 30 (93 cases;4.03%); 30 to 40 (598 cases;25.90%); 40 to 50 (1059 cases;45.90%); 50 to 65 (494 cases;21.40%); and over 65 (65 cases;2.82%).

**Table 1:** Characteristic of 2309 cervical cancer

Pathological type	Mean age (y)	total
Aquamous carcinoma	45.5	2106 (91.21%)
adenosquamous carcinoma	43.24	68 (2.94%)
adenocarcinoma	44.22	86 (3.72%)
Glass cell carcinoma	48	1 (0.04%)
Atypical carcinoid	45	1 (0.04%)
Squamous carcinoma combined glass cell carcinoma	40	1 (0.04%)
Squamous carcinoma combined neuroendocrine carcinoma	39	2 (0.09%)
Squamous carcinoma combined adenoid base cell cancer	57.67	3 (0.13%)
Scale-like basal cell carcinoma	43.5	2 (0.09%)
Lymphoepithelioma-like carcinoma	49	2 (0.09%)
villoglandular adeno-carcinoma	41.25	4 (0.17%)
neuroendocrine carcinoma	43.3	10 (0.43%)
clear cell carcinoma	51.25	4 (0.17%)
Minimal deviation adenocarcinoma	41.67	9 (0.39%)
Adenocarcinoma combined neuroendocrine carcinoma	33	3 (0.13%)
Adenocarcinoma combined clear cell carcinoma	65	1 (0.04%)
adenosquamous carcinoma combined neuroendocrine carcinoma	42.33	3 (0.13%)
Adenoid base cell carcinoma	51	2 (0.09%)
Adenoid cystic carcinoma	45	1 (0.04%)
Total	45.36	2309

**Figure 2:**
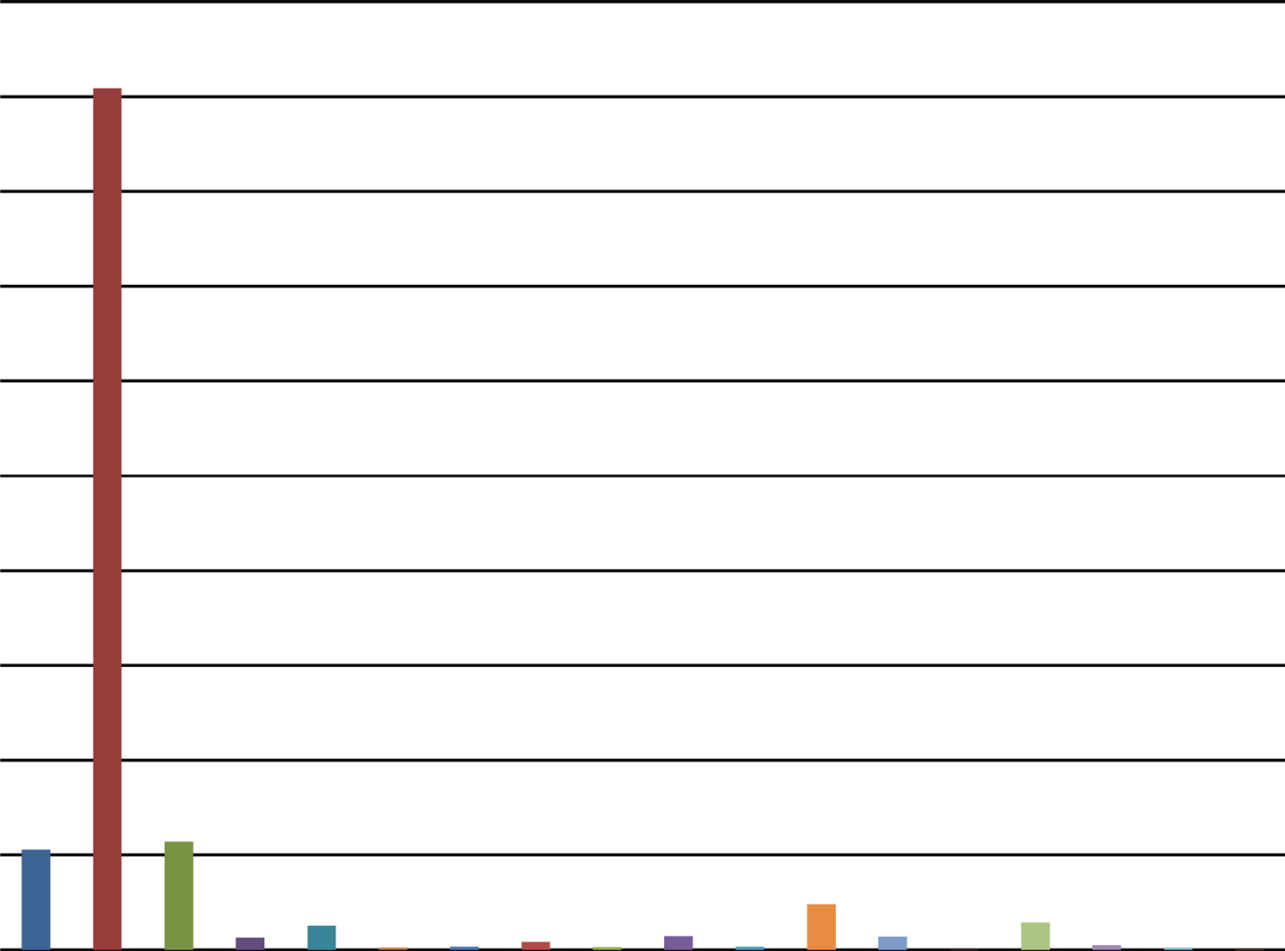
Distribution of HPV in cervical cancer

Table 2 and Figure 2 display the HPV infection results of the cervical cancer patients included in this study. The data reveals that 90.87% of the cervical cancer cases studied were currently infected with HPV, with 90.82% infected with high-risk HPV and 0.04% with low-risk HPV. The remaining 9.13% tested negative for HPV. The most common HPV subtypes among these patients were HPV16, HPV18, HPV58, HPV53, and HPV33. Their prevalence was 78.70% (1818/2309), 9.87% (228/2309), 4.16% (96/2309), 2.51% (58/2309), and 2.21% (51/2309), respectively. Of the 2309 cases, 230 (9.96%)were infected with multiple HPV subtypes; cases infected with both HPV16 and HPV18 (4.07% (94/2309)) and those with both HPV16 and HPV58 (1.82% (42/2309)) accounted for the largest proportions of this group.

**Table 2:** Prevalence and distribution of HPV in 2309 cervical cancer women

		Overall prevalence		Prevalence by age									
		All ages		≤ 30 y		30–40 y		40–50 y		50–65 y		≥ 65 y	
	Genotype	positive	Prevalence (%)	positive	Prevalence (%)	positive	Prevalence (%)	positive	Prevalence (%)	positive	Prevalence (%)	positive	Prevalence (%)
HR HPV types	**negative**	211	9.13	8	8.60	68	11.37	76	7.17	56	11.00	3	6.00
	**HPV16**	1818	78.70	72	77.42	465	77.76	855	80.66	385	75.64	41	82.00
	**HPV18**	228	9.87	9	9.68	64	10.70	100	9.43	51	10.02	4	8.00
	**HPV31**	26	1.13	2	2.15	3	0.50	13	1.23	7	1.38	1	2.00
	**HPV33**	51	2.21	1	1.08	14	2.34	26	2.45	9	1.77	1	2.00
	**HPV35**	5	0.22	0	0.00	2	0.33	1	0.09	2	0.39	0	0.00
	**HPV39**	7	0.30	1	1.08	2	0.33	3	0.28	1	0.20	0	0.00 2.00
	**HPV45**	17	0.74	0	0.00	1	0.17	15	1.42	0	0.00	1	
	**HPV51**	6	0.26	1	1.08	0	0.00	3	0.28	2	0.39	0	0.00
	**HPV52**	29	1.26	0	0.00	5	0.84	12	1.13	12	2.36	0	0.00
	**HPV56**	7	0.30	0	0.00	2	0.33	3	0.28	2	0.39	0	0.00
	**HPV58**	96	4.16	5	5.38	26	4.35	40	3.77	21	4.13	4	8.00
	**HPV59**	28	1.21	3	3.23	3	0.50	11	1.04	9	1.77	2	4.00 0.00
	**HPV26**	1	0.04	0	0.00	0	0.00	1	0.09	0	0.00	0	
	**HPV53**	58	2.51	0	0.00	54	9.03	4	0.38	0	0.00	0	0.00
	**HPV66**	10	0.43	0	0.00	3	0.50	4	0.38	3	0.59	0	0.00
	**HPV68**	5	0.22	0	0.00	0	0.00	5	0.47	0	0.00	0	0.00
													
LR HPV types	**HPV83**	1	0.04	0	0.00	0	0.00	1	0.09	0	0.00	0	0.00

**Figure 3:**
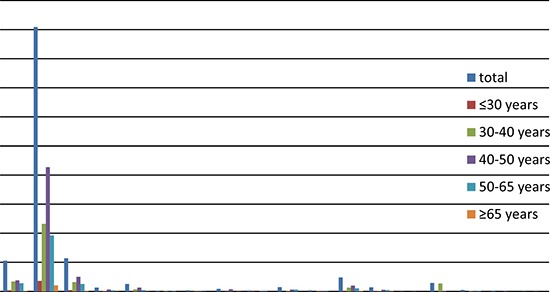
Distribution of HPV in cervical cancer by age

The age-specific prevalence of HPV among the cases was as follows: under 30: 91.40%; 30 to 40: 88.63%; 40 to 50: 92.83%; 50 to 65: 89%; and over 65: 94%. The three most prevalent subtypes were HPV16, HPV18, and HPV58, while the prevalence of the other subtypes differed between age groups. See Table 2 and Figure 3 for more details.

As can be seen from Table 4, among the 211 HPV-negative cases of cervical cancer, 171 (about 8.12%) tested negative for squamous cell carcinoma, 17 (25%) tested negative for adenocarcinoma, and 13 (15.12%) were negative for adenosquamous carcinomas. A high proportion of the cases diagnosed with special types of cervical cancer appeared to be HPV-negative. The percentages of HPV-negative cases in the cases of clear-cell carcinoma, minimal deviation adenocarcinoma, and denoid basal carcinoma were 75% (3/4), 33.33% (3/9), and 50% (1/2), respectively.

**Table 3:** Results of logistic analysis (*P* < 0.05 indicates that the type of pathology may be association with and the subtypes HPV persistent infection)

		squamous cell carcinoma		adenocarcinoma		adenosquamous carcinoma	
	Genotype	coefficient	*P*	coefficient	*P*	coefficient	*P*
HR HPV types	HPV16	1.424	<.0001	−1.237	<.0001	−1.226	< .0001
	HPV18	−1.608	<.0001	1.248	<.0001	1.586	< .0001
	HPV31	0.886	0.386	−13.059	0.986	−13.072	0.985
	HPV33	0.127	0.81	−0.424	0.677	−13.083	0.979
	HPV35	12.149	0.985	−11.05	0.986	−11.062	0.985
	HPV39	12.15	0.982	−12.051	0.989	−12.063	0.988
	HPV45	−1.484	0.006	0.731	0.481	1.742	0.007
	HPV51	−1.655	0.057	−11.05	0.985	−11.063	0.983
	HPV52	1	0.327	−13.06	0.986	−0.081	0.937
	HPV58	1.124	0.057	−0.368	0.612	−13.104	0.971
	HPV59	−0.223	0.718	0.203	0.843	0.7	0.346
	HPV56	−1.432	0.088	−12.051	0.989	1.47	0.176
	HPV26	10.147	0.984	−10.048	0.991	−10.061	0.99
	HPV53	8.34	0.929	−7.241	0.94	−7.254	0.932
	HPV66	−0.96	0.226	−12.052	0.987	−12.065	0.986
	HPV68	−0.957	0.393	−11.05	0.986	1.876	0.095
LR HPV types	HPV83	−14.384	0.972	−10.048	0.991	−10.061	0.99

**Table 4:** Distribution of HPV negative

Pathological type	total	negative
Aquamous carcinoma	2107	171 (8.12%)
adenocarcinoma	68	17 (25.00%)
adenosquamous carcinoma	86	13 (15.12%)
clear cell carcinoma	4	3 (75.00%)
Minimal deviation adenocarcinoma	9	3 (33.33%)
neuroendocrine carcinoma	10	1 (10%)
Adenocarcinoma combined clear cell carcinoma	1	1 (100.00%)
Adenoid base cell carcinoma	2	1 (50.00%)

**Figure 4:**
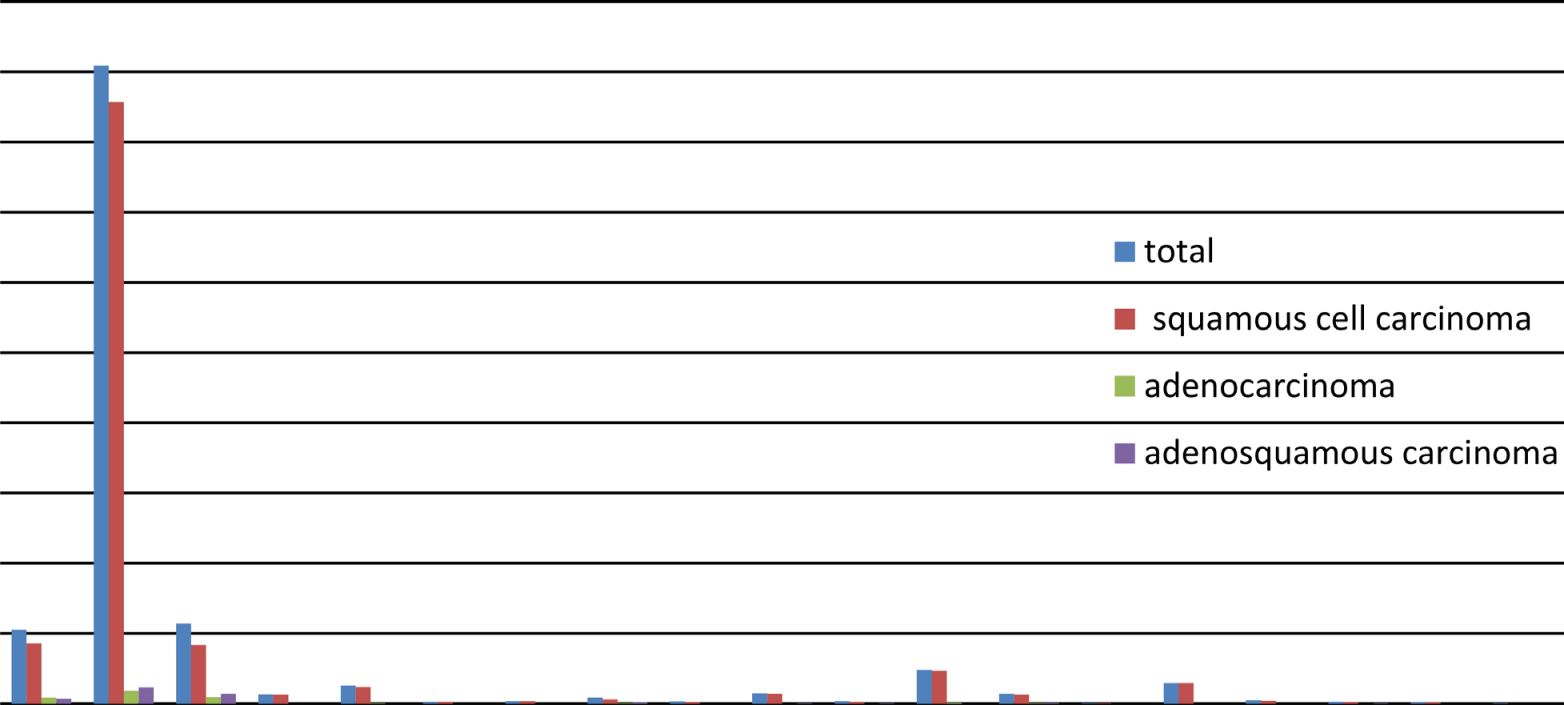
Distribution of HPV in cervical squamous cell carcinoma, cervical adenocarcinoma, and cervical adenosquamous carcinoma

The logistic regression analysis reveals that squamous cell carcinoma, adenocarcinoma, and adenosquamous carcinoma of the cervix were most closely correlated to persistent infection with HPV16 or HPV18, with *P* < 0.001. Adenosquamous carcinoma of the cervix may also be correlated with persistent HPV45 infection, with *P* = 0.007. Figure 4 and Table 3 show the detailed results of the analysis.

## DISCUSSION

A systematic survey and analysis of HPV infections among women in different regions can help effectively identify populations that are at a high risk of cervical cancer. Such information will ultimately provide an important basis for the prevention and treatment of cervical cancer as well as the development of HPV vaccines. HPV infection in cervical patients is different in different countries and in different regions [12, 13]. An American study revealed that the rate of high-risk HPV infection in cervical cancer was 94%, and in the study, 22 women (46.8%) were positive for HPV 16, 12 women (25.5%) were positive for HPV 18, and the remaining 13 women (27.7%) were positive for 12 other types of hrHPV [4]. An Indian study also determined that the rate of high-risk HPV infection in cervical cancer was 94% [14]. The most common HPV subtypes were determined to be HPV 16, HPV 18, HPV 31, HPV 33, and HPV 35. However, these results were obtained fromsmall sample studies.

In contrast, we obtained different results. This study analyzed HPV infections among 2309 cervical cancer patients.90.87% of the cases in this study were currently infected with HPV, with 90.82% infected with high-risk HPV types. The most common subtypes among these cases were HPV16, HPV18, HPV58, HPV53, and HPV33.

High-risk HPVs are known to be the primary cause of cervical cancer. HPV prevalence varies with the grades of cervical cancer and cervical precancerous lesions as well as differs between regions and ages. Xiao Meizhu *et al*. [15] analyzed HPV infections among 2817 cases of cervical intraepithelial neoplasia (CIN) in Beijing. They found that high-risk HPV had infected 477 (78.2%) of the 610 CIN1 cases, with HPV16, HPV58, HPV52, and HPV18 being the most prevalent subtypes. Furthermore, 2060 (93.3%) of the 2207 CIN2 cases were infected with high-risk HPV, and HPV16, HPV58, HPV33, and HPV52 were the most frequently observed. Krishnan Baskaran et al. [14] estimated that the prevalence of high-risk HPV is as high as 94% among cervical cancer cases, which is significantly higher than that among the general population and women with cervical precancerous lesions. HPV16, HPV18, HPV45, HPV58, and HPV31 have been demonstrated to be the most prevalent HPV subtypes among cervical cancer cases.

A few studies have suggested that persistent infections with different HPV subtypes may be responsible for different histological types of cervical cancer, and the corresponding prognosis tends to vary. In this study, a logistic regression analysis was conducted to determine whether there is a link between the occurrence of different histological cervical cancer types and specific HPV subtypes. The results show that squamous cell carcinoma, adenocarcinoma, and adenosquamous carcinoma of the cervix areclosely correlated with a persistent infection with HPV16 or HPV18, with *P* < 0.001. Adenosquamous carcinoma of the cervix may also be correlated with a persistent HPV45 infection, with *P* = 0.007. The results suggest that some special histological types of cervical cancer may be associated with infection by certain HPV subtypes. However, due to the small sample size, further research based on a large sample is needed in order to validate these findings. These findings may provide a new approach for the prevention, screening, and treatment of cervical cancer.

HPV has been proven to be the primary cause of cervical lesions, however, it is not the only cause. The occurrence and development of cervical lesions result from the combined effect of multiple factors. Furthermore, 9.13% of the cervical cancer cases included in this study tested negative for HPV, with squamous cell carcinomas, adenocarcinomas, and adenosquamous carcinomas accounting for the majority of these cases. Research has shown that an estimated 80% of women get infected with HPV at some point in their lifetime; however, most of these cases are transient infection. Most HPV infections only last for 8 to 24 months before the immune system clears them. Nonetheless, in very few cases, the infections tend to persist and progress into lesions [16]. These cases will test negative for HPV because the viruses will ultimately be killed by the body's immune system. However, despite the negative test results, the cervical lesions will last for a long period of time.

Similar to the previous study, HPV-negative cases made up a relatively high percentage of the women with special histological types of cervical cancer, and the percentage was higher among the cervical adenocarcinoma cases than among the squamous cell carcinoma cases. In an observational study of 136 cervical cancer patients, Rodriquez-Carunchio L. *et al*. found that the percentage of HPV-negative cases in cervical adenocarcinoma patients was 15.6% as compared to 2.9% of squamous cell carcinomas cases [17]. The cervical adenocarcinomas that were unrelated to HPV infection include some uncommon subtypes, such as clear-cell carcinoma, mesonephric adenocarcinoma, endometrioid adenocarcinoma, and minimal deviation adenocarcinoma of the cervix [18]. In a study by Pigor *et al*., HPV-DNA was detected in 91% of mucinous adenocarcinomas (including mucinous adenocarcinoma of the cervical canal, intestinal-type, and endometrioidmucinous adenocarcinoma) and in all adenosquamous carcinoma cases. In contrast, no HPV-DNA was detected in the non-mucinous adenocarcinoma cases (including 4 clear-cell carcinomas, 1 serous carcinoma, 1 mesonephric adenocarcinoma, and 2 minimal deviation adenocarcinomas). The above data demonstrate that some common types and rare subtypes of cervical adenocarcinomas are uncorrelated with HPV infection [19]. Perhaps cervical lesions result from other causes. There may be high-risk HPV we have not yet found, perhaps due to the limitations of HPV detection methods that result in false negative. The specific reasons need to be further studied.

The study has limitations. Although the sample size is large, the study was only a single-center retrospective study. Some special types of cervical cancer are relatively small, which can affect the accuracy of the results of the logistic regression analysis. A logistic regression analysis was used to determine whether there is a link between the occurrence of different histological cervical cancer types and specific HPV subtypes, and it was found that there is an association between cervical cancer and persistent infection with HPV16 or HPV18. Nonetheless, it is very difficult to define the case with persistent infection. A large-scale multicenter prospective study is to be needed to confirm the results.

The results of this study show that HPV16 and HPV18 were the most prevalent HPV subtypes in cervical cancer patients across all age groups in West China, followed by HPV58, HPV53, and HPV33. The prevalence of the HPV subtypes, excluding HPV16 and HPV18, varied significantly between the age groups. Additionally, 9.13% of the cases included in this study were not associated with HPV. Most of these cases were diagnosed with special types of cervical cancer. This suggests that HPV testing without the use of cytology may overlook some special types of cervical cancer that account for approximately 10% of all cervical cancer cases. Therefore, clinical attention should be paid to this finding.

## MATERIALS AND METHODS

### Study design

This is a retrospective study. The West China Second University Hospital of Sichuan University is known as the largest gynecological cancer prevention and treatment center in West China. This study focused on women with pathologically confirmed invasive cervical cancer who received treatment between June 2011 and January 2016 at this hospital. Two pathologists reexamined pathological sections of paraffin-embedded tissues from the cervical cancers. If the two pathologists had inconsistent views, then a third pathologist was consulted to resolve the issue. Patients with uterine sarcoma, metastatic cervical cancer, or other malignant tumors associated with the cervix were excluded from this study. Because the study is a retrospective study, there is no ethical committee approval. This is in accordance with institutional or national policies concerning research approvals.

### HPV testing

Five 10 μm thick sections were cut from each paraffin-embedded tissue specimen, and these sections were then placed into 1.5 mL centrifuge tubes. Next, 1 mL of xylene was added to each centrifuge tube and blended with the tissues by oscillation at room temperature. After centrifugation, the supernatant was removed, and 1 mL of absolute ethanol was added to the tubes to eliminate the xylene. Subsequently, a kit (Qiagen RNA/DNA Mini kit) for purifying DNA from tissue produced by Qiagen was used to extract DNA from the specimens according to the kit handbook. Afterwards, the extracted DNA was dissolved in 50 μL of elution buffer and stored at −20°C for use.

The DNA purified from the tissues was placed in an HPV genotyping kit (Human papillomavirus nucleic acid typing detection kit (flow fluorescence hybridization) produced by Shanghai Tellgen Corporation). There were 27 HPV subtypes of interest, including 17 high-risk subtypes (HPV-16, 18, 26, 31, 33, 35, 39, 45, 51, 52, 53, 56, 58, 59, 66, 68, and 82) and 10 low-risk subtypes (HPV-6, 11, 40, 42, 43, 44, 55, 61, 81, and 83). The hybridization products were tested using a Luminex 200, which is a multifunctional suspension array system.

### Statistical analysis

The data from the test was analyzed using the statistics package SPSS 22.0. The measurement data, expressed as x ± s, were tested through a *t*-test. The enumeration data, expressed in %, were tested using the χ2 test. A logistic regression analysis was performed to examine the relationships between the different histological types of cervical cancer and the HPV subtypes. *P*-values were calculated and used to determine whether a certain HPV subtype had an influence on a particular histological type of cervical cancer. In this study, α = 0.05 was taken as the standard level of significance; thus, the difference between data is considered statistically significant when *P* < 0.05.
